# Effect of UV Irradiation and TiO_2_-Photocatalysis on Airborne Bacteria and Viruses: An Overview

**DOI:** 10.3390/ma14051075

**Published:** 2021-02-25

**Authors:** Nina Bono, Federica Ponti, Carlo Punta, Gabriele Candiani

**Affiliations:** 1 GenT LΛB & µBioMI LΛB, Department of Chemistry, Materials and Chemical Engineering “G. Natta”, Politecnico di Milano, Via L. Mancinelli, 7, 20131 Milan, Italy; nina.bono@polimi.it (N.B.); federica.ponti@polimi.it (F.P.); 2 Laboratory for Biomaterials and Bioengineering, Canada Research Chair I in Biomaterials and Bioengineering for the Innovation in Surgery, Department Min-Met-Materials Engineering, Research Center of CHU de Quebec, Division of Regenerative Medicine, Laval University, Quebec City, QC G1V 0A6, Canada; 3 OSCMLab, Department of Chemistry, Materials and Chemical Engineering “G. Natta”, Politecnico di Milano, Via L. Mancinelli, 7, 20131 Milan, Italy; carlo.punta@polimi.it; 4 Milano Politecnico Research Unit, National Interuniversity Consortium of Materials Science and Technology—INSTM, Via Mancinelli 7, 20131 Milan, Italy

**Keywords:** UV light, titanium dioxide, photocatalysis, disinfection, antibacterial, antiviral

## Abstract

Current COVID-19 pandemic caused by the severe acute respiratory syndrome coronavirus 2 (SARS-CoV-2) has put a spotlight on the spread of infectious diseases brought on by pathogenic airborne bacteria and viruses. In parallel with a relentless search for therapeutics and vaccines, considerable effort is being expended to develop ever more powerful technologies to restricting the spread of airborne microorganisms in indoor spaces through the minimization of health- and environment-related risks. In this context, UV-based and photocatalytic oxidation (PCO)-based technologies (i.e., the combined action of ultraviolet (UV) light and photocatalytic materials such as titanium dioxide (TiO_2_)) represent the most widely utilized approaches at present because they are cost-effective and ecofriendly. The virucidal and bactericidal effect relies on the synergy between the inherent ability of UV light to directly inactivate viral particles and bacteria through nucleic acid and protein damages, and the production of oxidative radicals generated through the irradiation of the TiO_2_ surface. In this literature survey, we draw attention to the most effective UV radiations and TiO_2_-based PCO technologies available and their underlying mechanisms of action on both bacteria and viral particles. Since the fine tuning of different parameters, namely the UV wavelength, the photocatalyst composition, and the UV dose (viz, the product of UV light intensity and the irradiation time), is required for the inactivation of microorganisms, we wrap up this review coming up with the most effective combination of them. Now more than ever, UV- and TiO_2_-based disinfection technologies may represent a valuable tool to mitigate the spread of airborne pathogens.

## 1. Introduction

Since the outbreak of respiratory disease in December 2019 and subsequent COVID-19 pandemic caused by the novel coronavirus SARS-CoV-2 (severe acute respiratory syndrome coronavirus 2), great effort has been devoted to the development and widespread use of antimicrobial technologies to mitigate airborne microbial contaminations [1]. Broadly speaking, disease-causing bacteria (e.g., *Escherichia coli*, *Staphylococcus aureus*, and *Pseudomonas aeruginosa*) and viruses (e.g., swine influenza H1N1 and human coronaviruses) and overall airborne pollutants have attracted considerable attention so far and spurred the development of a number of purification technologies and disinfection protocols [2,3]. Environmental pollution, and airborne contamination specifically, is a complex and intriguing issue that involves the presence of contaminants in the form of particulate matter, non-volatile biological agents such as bacteria, molds, and viruses transferred by the bioaerosols, and inorganic gaseous pollutants such as NO_x_, SO_x_, CO, CO_2_, and volatile organic compounds (VOCs) [4]. Industry has done well enough throughout the years to improve disinfectant and sanitizing chemistries. However, the lack of time, resources, and budgets necessary to stay ahead of ever-growing public health threats continues to pose challenges.

Air sanitation technologies are thus aimed at trapping and inactivating pollutants, i.e., potential pathogens and parts thereof. Such systems and devices rely on the use of disinfection and sterilization technologies. Broadly speaking, disinfection describes the process of complete elimination of vegetative forms of (micro)organisms except the bacterial spores from inanimate objects. Instead, sterilization describes the process of destruction or elimination of all microbial life forms (i.e., both vegetative and spore forms), which is basically carried out by physical or chemical methods. Another term frequently used with reference to sanitation is the word cleaning (also called cleansing), which refers to the removal of visible soil (i.e., organic and inorganic matter) from objects and surfaces, and normally it is accomplished manually or mechanically using water with detergents or enzymatic products [5,6,7].

Widely used air sanitation technologies include (i) treatment with non-thermal plasma (NTP), (ii) thermal treatment, (iii) use of antimicrobial materials-embedded filters, (iv) ultraviolet (UV) light irradiation, and (v) the use of photocatalysts [3,4,8]. In the NTP cleaning systems, energetic electrons excite, dissociate, and then ionize gas molecules, giving rise to chemically reactive oxygen species (ROS) such as atomic oxygen, hydroxyl radicals, and ozone [8]. Even if such active species are known to inactivate biotic and abiotic particles, concerns about the formation of environmentally harmful secondary pollutants, such as ozone, CO, or NO_2_ are limiting the widespread use of such treatments [9,10]. In thermal treatments, exposure to high temperature induces protein denaturation through disrupting the polypeptide structures, thus causing irreversible damages to microorganisms [11]. Besides, although thermal treatments were found very promising, they are very energy-consuming and therefore costly [12]. Filtration systems, in which airborne biological particles are collected on the surface of a filter, are great options to overcome the limitations of the aforementioned technologies. However, these antimicrobial materials-embedded filters are generally effective in the short-term because of the accumulation of dust that progressively clog them and cause a large pressure drop, so that they must be replaced regularly to prevent the possible re-introduction of airborne microorganisms into the environment all of a sudden [9].

Another effective approach to prevent the spread of airborne-mediated microbial diseases relies on the inactivation of harmful bacteria and viruses by means of UV light [13,14,15]. This is a more cost-effective and ecofriendly option than using other widespread technologies. From a practical point of view, a UV lighting system is easy to be installed and requires very little energy expenditure as compared to thermal treatments. In this light, UV lamps are often installed in the ceiling of surgery rooms in hospitals and health care facilities in order to inactivate nearby bioaerosols [16]. Of note, UV germicidal irradiation has been found very suitable to inactivate any kind of microorganism, such as drug-sensitive and even multi-drug-resistant bacteria [17] and different viral strains [18,19,20,21,22,23]. Such antimicrobial activity has been ascribed to the damaging effect of UV light on the biopolymers they are made of; it is critically dependent on the type of microorganism and operating conditions, i.e., the UV wavelength, the UV intensity, and the irradiation time, and it is a function of some environmental conditions (i.e., temperature and relative humidity) [24,25]. Unfortunately, the use of germicidal UV lighting systems in public space is limited because conventional UV light sources may also be threatening to human health [26,27].

It has also been shown that some microorganisms can recover from sub-lethal UV-irradiation through a photoenzymatic process called photoreactivation [28]. To overcome this issue, UV light is often used in combination with photocatalytic materials, also known to as photocatalysts [29,30], such that UV photocatalytic oxidation (PCO) has become the most exploited air sanitation technology [31,32,33]. Of note, PCO shows many advantages as compared to other technologies, including the ability to treat complex mixtures of different pollutants simultaneously, being relatively cheap, and easy to maintain and operate [2,34,35,36].

The purpose of this literature review is to thoroughly discuss the underlying mechanism and applications of the antimicrobial activity exerted by the UV light *per se* and used in combination with photocatalytic materials, with a main focus on the widely used titanium dioxide (TiO_2_) nanoparticles (NPs). Since the fine tuning of different parameters, namely, the UV wavelength, the photocatalyst composition, and the UV dose (viz, the product of UV light intensity and the irradiation time), is required for an inactivation of microorganisms, we wrap up this review coming up with the most effective combination of them. Our aim is to provide the readers with a comprehensive literature survey pinpointing a cause–effect relationship between the irradiation patterns and parameters and the ultimate effect on microorganisms. Such information would be very helpful to design next-generation air disinfection technologies.

## 2. UV-Induced Disinfection

### 2.1. Mechanism of Action

In recent years, UV light irradiation has been extensively investigated as a powerful mean of disinfection or sterilization [30,37,38].

It is worth noting that the inactivation of microorganisms is strictly dependent on the amount of radiation absorbed and capable of giving rise to detrimental effects. Accordingly, some parameters play a key role on the disinfection behavior, one above all the UV dose (referred to as fluence), generally expressed as the product of the UV light intensity (I) and the irradiation time (T_irr_), according to Equation (1):

(1) $${\mathrm{UV}\;\mathrm{dose}=I\times T}_{\mathrm{irr}}$$

where UV dose is commonly expressed in J cm^−2^ = W s cm^−2^.

As a rule of thumb, there are a couple of mechanisms through which the UV radiation can damage microorganisms: (i) the photo-induced reactions resulting from the direct absorption of UV photons by biopolymers, especially nucleic acids (NAs) and proteins, which are the basic constituents in common between bacteria and viruses [39,40], and (ii) the photo-oxidation triggered by ROS generated after UV irradiation of exogenous and endogenous photosensitizers, i.e., powerful oxidant materials or photosensitive molecules other than NAs and proteins [41].

Depending to the radiation wavelength, the UV light is categorized as UV-A (λ = 315–400 nm), UV-B (λ = 280–315 nm), and UV-C (λ = 100–280 nm). It is widely accepted that UV-C and UV-B light are the most effective in inducing DNA and RNA photoproducts. This is because NAs contain monosaccharides and nucleobases, which have a maximum absorption peak at 200 and 265 nm, respectively. Conversely, near UV-A light was found to be less effective in triggering NAs damage, as it is not directly absorbed by such biopolymers [42,43,44]. Nonetheless, UV-A radiations were found to induce photo-oxidation through the generation of free radicals and ROS, that, in turn, may ultimately damage NAs, proteins, and lipids [39,42,45].

Overall, the UV-induced microbial inactivation is the result of photoreactions occurring because of the photon absorption by NAs [46]. Indeed, there exists a causal relationship between the wavelength of the UV beam (at a given intensity) and NAs damage [47]. The mechanism of direct NAs damage induced by UV irradiation is outlined herein below.

#### 2.1.1. Direct NAs Damage by UV Irradiation

The UV-induced damage of DNA and RNA molecules mostly occurs by means of UV-B and UV-C radiation and over wavelengths spanning from 200 to 300 nm [20,21]. 

UV light has been found to trigger the generation of photoproducts that may lead to structural and functional changes of NAs and to defects to the replication/transcription/translation machinery [47]. Therefore, UV light is potentially lethal to a wide variety of organisms, spanning from prokaryotes to plants and even animals [42]. Besides, when used for viral disinfection, UV light has been found to inhibit genome replication and infection, ultimately leading to the inactivation of the viral particle [48]. 

**Figure 1:**
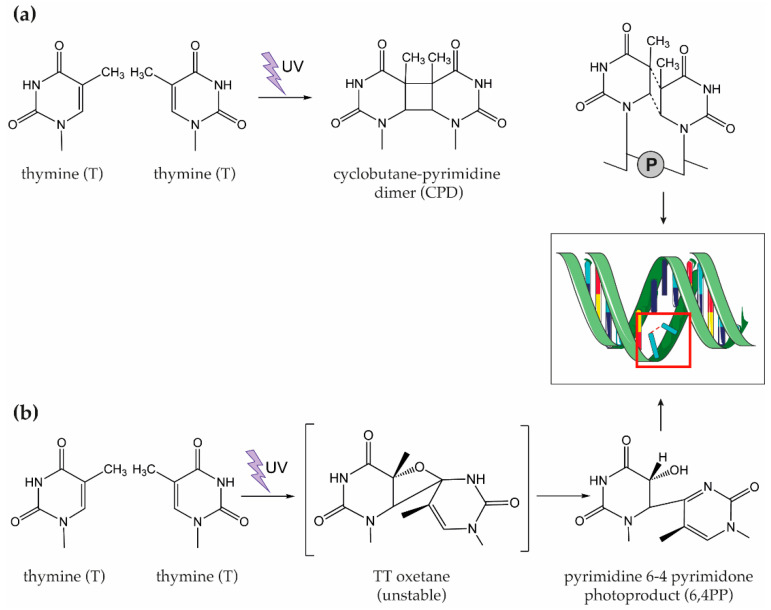
Schematic representation of pyrimidine dimers formation after DNA exposure to UV light. Formation of (**a**) cyclobutane pyrimidine dimers (CPDs) and (**b**) pyrimidine-6,4-pyrimidone photoproducts (6,4PP) between two adjacent thymine (T) nitrogenous bases. In the case of RNA, similar reactions may occur for uracil (U).

The most studied and best described UV-induced photoreaction between and within NAs results in the covalent linkage of two spatially adjacent pyrimidine bases (i.e., thymine (T) in DNA, uracil (U) in RNA, and cytosine (C) in both DNA and RNA) to give rise to photodimers named cyclobutene pyrimidine dimers (CPDs) and pyrimidine-6,4-pyrimidone (6,4PP) photoproducts, together with the Dewar valence isomers [42,47,49,50]. Figure 1 depicts the formation of pyrimidine dimers after exposure of DNA to UV light. Once generated, CPD and 6,4PP dimers are responsible for the distortion of the double helix through a bending of 7–9° and 44°, respectively [42]. Such modifications are responsible for the blockade of the DNA replication [51].

It is apparent that the composition of NAs (i.e., the exact sequence of nucleotides in the molecule) thus plays a major role in the formation of photodimers as a consequence of UV exposure. Of note, even though pyrimidine photoproducts represent the largest photochemical modification of double-stranded (ds) DNA, purines display some photoreactivity as well. For instance, if the purine base adenine (A) in close proximity to another A or a T is UV-irradiated, A-A or A-T photoactivated dimers may also form.

Besides, it was found that the shorter the NA sequence, the greater the probability to be severely affected by UV radiation [52]. In this regard, the pyrimidine dimers T-T and T-C were found more photoreactive than C-C and C-T analogues [53,54,55], and single-stranded NAs (ssDNA and ssRNA) were more sensitive to UV exposure than the double-stranded counterparts. Moreover, regardless of the number of strands, DNA is less prone than RNA to UV-induced damage [43,56]. Nevertheless, the effects of UV light on RNA have been much less investigated.

#### 2.1.2. Oxidative Damage to NAs and Proteins

In addition to the direct effect of UV radiation on NAs, the oxidative damage of DNA and RNA may arise as a result of other UV-triggered reactions. In this context, the generation of ROS after the absorption of UV-A light by any photosensitive molecules other than NAs may lead to the oxidation of the guanine (G) base, thus giving rise to 8-oxo-7,8-dihydro-2′-deoxyguanosine (8-oxodGua) adduct [47,50]. If not properly repaired by cellular self-repair mechanisms (for additional information on this topic, please refer to [49,50]), such UV-induced NA damages may impact the overall NAs structure and function, and impair protein translation, such that cells ultimately undergo death [51,57].

On the other hand, proteins (i.e., the main component of prokaryotic and eukaryotic cells and the viral capsid) are another target of photo-oxidation [58,59]. Indirect photochemical damage consists in the absorption of light by sensitizers and the electron transfer to the molecular oxygen (O_2_) dissolved in fluids. This gives rise to the formation of ROS, such as the singlet oxygen (^1^O_2_) and hydroxyl radical (OH•), which, in turn, may react with the side chain of some given amino acid residues (e.g., Trp, His, Tyr, Met, and Cys) leading to protein oxidation [40,60,61]. This phenomenon may eventually cause proteins to lose their structure and function, and become very harmful to microorganisms. Besides, it is worth noting that oxidized proteins are poorly repaired because they become less (or sometimes more or too) susceptible to proteolysis [61].

### 2.2. Antimicrobial Effects of UV Light

Germicidal UV-C radiation proved to efficiently inactivate airborne bacterial pathogens. Based on literature data, the UV-C dose needed to inactivate airborne pathogens on surfaces is usually in the order of tens to hundreds mJ cm^−2^ [17,62,63]. Even more interestingly, it has been shown that far UV-C radiation (λ = 207–222 nm; UV dose = 135 mJ cm^−2^) does efficiently inactivate drug-resistant bacteria, and without apparent harm to mammalian skin [64,65]. These are very surprising results if considering that UV-C radiation is known to be harmful to microorganisms, such as viruses, bacteria, yeasts, and fungi within seconds, but it also causes skin irritation and severe eye damage [37]. 

The UV radiation has been reported to be an efficient mean to inactivate viral particles as well. Using a conventional UV-C lighting system (λ = 254 nm) capable of providing an UV dose of 1.1 mJ cm^−2^, McDevitt et al. found an inactivation of ~95% of airborne influenza virus H1N1 [66]. Similar results were reported by Welch et al. when using a far UV-C light (i.e., λ = 207–222 nm) and an irradiation dose as low as 2 mJ cm^−2^ [67]. UV irradiation has also been envisioned for the inactivation of human coronaviruses. As an example, SARS-CoV virus was found to be efficiently inactivated when exposed to an UV-C light of 3.6 J cm^−2^ for 15 min [15] or an irradiation dose as low as 7 mJ cm^−2^ [68], while Bedell and colleagues reported the complete inactivation of MERS-CoV after a 5-min exposure to UV-C light [69]. 

In the context of the current COVID-19 pandemic, the whole scientific community was asked to accelerate technological advances critical to face the spread of the virus. Accordingly, the results have come faster than ever before, while other research is under way. Different studies have outlined the inactivation of SARS-CoV-2 through the application of the UV-C radiation, and pointed to doses of 2.6 J cm^−2^ [70], 16.9 mJ cm^−2^ [71], and 1 J cm^−2^ [72]. Data on other human coronavirus strains have laid the basis for such breakthroughs. In this regard, UV-C doses of 1.7 and 1.2 mJ cm^−2^ (λ = 222 nm) were found effective in inactivating HCoV-229E and HCoV-OC43 viruses, respectively. Due to inherent similarity between these coronaviruses and the etiological agent of COVID-19, the authors speculated that the same doses may be used to inactivate SARS-CoV-2 [73]. 

## 3. UV-Activated Photocatalysis

### 3.1. Operating Principles

**Figure 2:**
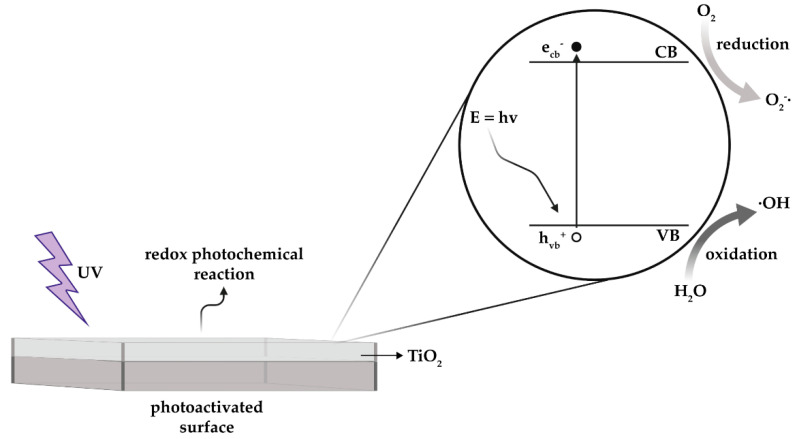
Schematic representation of a photoactivated surface and the mechanism of photocatalysis of a semiconductor (SC). A SC is characterized by a relatively low energetic band gap between the lower entirely occupied valence band (VB) and the higher unoccupied conduction band (CB). The adsorption of a photon by the SC, with a minimum energy at least equal to the band gap, promotes the excitation of an electron (e^−^) from the VB to the CB.

In recent years, we have witnessed a second surge of interest in photocatalysis by means of semiconductors (SCs) as a green and ecofriendly method to degrade a vast array of pollutants and biological agents specifically. Even if the photocatalytic mechanism strongly depends on the kind of pollutant, interfacial redox reactions of electrons (e^−^), and holes that are generated when the SC catalyst is exposed to a light of sufficient energy, are the primary reactions underlying the photocatalytic mechanism (Figure 2). The photocatalytic process relies on the excitation of an e^−^ from the valance band (VB) of the photocatalytic material to the conduction band (CB) when exposed to UV light (i.e., photon energy (E) = hʋ), thereby leaving a positive hole (h^+^) in the VB (Figure 2). 

Among the array of photocatalysts used for disinfection purposes, which includes zinc oxide (ZnO), tungsten oxide (WO_3_), and TiO_2_, the latter metal oxide is by far the most researched catalyst so far. Its great photoactivity, stability, cost-effectiveness, and nontoxic nature, together with the possibility to be used at ambient temperature and pressure, are just a few of the many advantages exploited by this material used in combination with UV lighting. Moreover, the ability of TiO_2_ to induce the photocatalytic degradation of almost any kind of organic and living pollutants, including bacteria and viruses, has fostered its use in purification technologies [74,75,76,77,78,79,80].

Noteworthy, the photocatalytic activity of TiO_2_ strongly depends on the material structure, as the distinct crystalline phases may lead to differences in the extent of recombination of e_cb_^−^ and h_vb_^+^. Indeed, TiO_2_ exhibits three main polymorphic forms, namely, anatase, rutile, and brookite. Although both anatase and rutile phases have been extensively used as photocatalysts, anatase has long been considered the most photoactive TiO_2_ type [81,82]. Nevertheless, studies have shown that anatase–rutile mixtures were more effective photocatalysts than pure anatase [83]. In this context, Degussa P25 nanopowder is a widely used, commercially sourced titania photocatalyst that contains approximately 85–70% anatase and 15–30% rutile crystallites. Actually, it is not easy to find a photocatalyst showing activity greater than that of P25, and it has, therefore, been used as a *de facto* standard TiO_2_ photocatalyst.

TiO_2_ photocatalysts generate strong oxidizing power when illuminated with UV light with wavelengths of less than 385 nm [84]. On the other hand, doping TiO_2_ with either N, C, S, or metals such as Sn, Pd, and Cu has been found to extend the wavelength spectrum of radiation absorption, so that also visible light can be used to trigger photocatalysis [85]. Generally speaking, photocatalytic TiO_2_ can be used in the form of (i) powder (e.g., Degussa P25), usually dispersed in aqueous solutions, (ii) film/coating applied to various substrates, or (iii) immobilized on surfaces [86,87,88,89,90,91].

As the photocatalytic inactivation of contaminants is a synergistic antimicrobial effect of UV light and oxidative radicals generated at the irradiated TiO_2_ surface, the dose of radiation administered is a key parameter when the UV light, whether or not used in combination with TiO_2_, is used as an antimicrobial mean.

### 3.2. Photocatalysis-Induced Damage by ROS Formation

When TiO_2_ is used as the SC, the following reaction occurs (Equation (2)):

(2) $${\mathrm{TiO}}_{2}+\mathrm{hv}\to {e_{\mathrm{cb}}}^{-}+{h_{\mathrm{vb}}}^{+}$$

The e^−^ are then free to migrate within the CB, while the h^+^ may be filled by the migration of an e^−^ from an adjacent molecule, leaving the latter with a hole, so that the process may be repeated at the surface of the photocatalyst. The e^−^ and h^+^ may fast recombine in a non-productive reaction (i.e., bulk recombination). More interestingly, when in contact with O_2_ and water (H_2_O) present in the environment (e.g., humid air), they may undergo charge trapping, with these two molecules acting as e^−^ and h^+^ scavengers, respectively. The result is the formation of superoxide anion (O_2_^−^•) (Equation (3)) and hydroxyl radical (•OH) (Equation (4)), respectively, as first ROS.

(3) $$O_{2}+{e_{\mathrm{cb}}}^{-}\to {O_{2}}^{-}•$$

(4) $${h_{\mathrm{vb}}}^{+}+H_{2}O\to •\mathrm{OH}+{H_{\mathrm{aq}}}^{+}$$

Moreover, when exposed to humid air, these species can further react. Specifically, O_2_^−^• leads to the formation of a hydroperoxyl radical (•OOH) (Equation (5)), while •OH undergoes fast coupling with another transient •OH, thus affording hydrogen peroxide (H_2_O_2_) (Equation (6)). Nevertheless, once formed in the presence of the activated SC, H_2_O_2_ acts as an e^−^ scavenger, thereby promoting once again the formation of •OH (Equation (7)).

(5) $${O_{2}}^{-}•+H^{+}\to •\mathrm{OOH}$$

(6) $$•\mathrm{OH}+•\mathrm{OH}\to H_{2}O_{2}$$

(7) $$H_{2}O_{2}+{e_{\mathrm{cb}}}^{-}\to •\mathrm{OH}+{\mathrm{OH}}^{-}.$$

**Figure 3:**
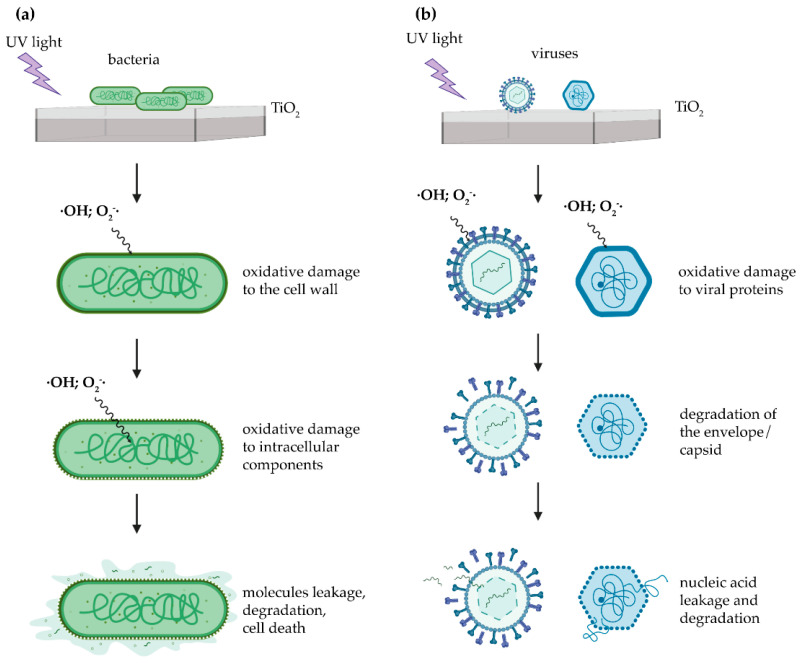
Mechanism of photocatalytic inactivation of (**a**) bacteria and (**b**) viruses. (**a**) Reactive oxygen species (ROS), namely, hydroxyl radicals (•OH) and superoxide anions (O_2_^−^•), generated after UV-irradiation of TiO_2_ first damage the cell wall layers, thus allowing the leakage of small molecules such as ions. ROS can thus further penetrate the cell, such that the degradation of the internal components may occur, followed by complete mineralization. The degradation process may occur progressively from the side of the cell in contact with the catalyst. (**b**) •OH and O_2_^−^• generated at the UV-activated TiO_2_ surface are able to degrade the capsid and envelope proteins, and phospholipids of non-enveloped and enveloped viruses, respectively. Besides, the leakage and consequent NAs degradation occurs, ultimately leading to the inactivation of the viral particles. Image is created with BioRender.com.

The fast reaction of such oxygen-centered radicals (i.e., •OOH, •OH, and O_2_^−^•) with organic molecules found in microorganisms (i.e., proteins and, above all, polyunsaturated fatty acids (PUFA)) represents the first step in the antimicrobial process, as described below. Of note, ROS act by promoting hydrogen atom transfer (HAT) reactions from the organic substrates (R-H), such that new carbon-centered radicals (R•) are formed (Equation (8)). In turn, these radicals may react with O_2_ and promote a radical chain (Equations (9) and (10)) that ultimately lead to the complete mineralization (Figure 3) (Equation (11)), i.e., the total oxidation of the substrate [92,93].

(8) $$•\mathrm{OH}+\text{R-H}\to R•+\;H_{2}O$$

(9) $$R•+O_{2}\to {\mathrm{RO}}_{2}•$$

(10) $${\mathrm{RO}}_{2}•+\text{R-H}\to \mathrm{ROOH}+R•$$

(11) $$•\mathrm{OH}+\mathrm{Organic}+O_{2}\to {\mathrm{CO}}_{2},\;H_{2}O.$$

Generally speaking, the UV photocatalytic inactivation of bacteria is mainly due to damages of the cell wall, membrane, intracellular enzymes, and NAs induced by ROS and their by-products [94]. Sunada et al. proposed a three-step mechanism for the photokilling of bacteria by irradiated TiO_2_-surfaces (Figure 3a): (i) attack of the cell wall by ROS, (ii) disordering of the inner cytoplasmic membrane, and (iii) degradation of the intracellular components [95]. Specifically, although the peptidoglycan layer of the cell wall was found to be slightly susceptible to the attack of ROS, the peroxidation of lipids of the lipopolysaccharide layer (LPS) present in Gram-negative bacteria and the PUFA found in both Gram-positive and Gram-negative bacteria are considered the main targets. Once the cell wall becomes permeable, •OH and, to a lesser extent, O_2_^−^• pass through the pores and gain access to the cytoplasm, such that they may damage NAs and coenzyme-A [96,97,98,99]. 

In the case of viruses, •OH and O_2_^−^• were suggested to be responsible for the degradation of organic compounds, such as the phospholipid bilayer and the envelope (in enveloped viruses) and capsid proteins, such that the leakage and consequent degradation of the genetic material may occur (Figure 3b) [100,101,102].

### 3.3. Antimicrobial Effects of UV Light-Induced TiO_2_ photocatalysis

The antibacterial activity of UV-irradiated TiO_2_ was first demonstrated by Matsunaga and coworkers in 1985 [103]. Since then, a substantial body of literature has showcased the antimicrobial effects of photocatalytic TiO_2_ on both Gram-negative and Gram-positive bacteria. Of note, the determination of the antimicrobial activity of photocatalytic materials is typically assessed in aqueous media or in dry conditions, as described in dedicated ISO norms (e.g., ISO 15714:2019 and ISO 27447:2009 [104,105]) and in a number of studies herein reported. However, it is worth noting that bench testing the antimicrobial effectiveness of materials and devices in aqueous media is far from the real-world scenario where microorganisms are airborne. As a result, the significance of these findings might be questionable.

The most relevant examples of TiO_2_ nanoparticles and microparticles used in the form of powder, typically dispersed in water (Table 1), or immobilized onto surfaces (Table 2) are reported in the tables herein below. Likewise, UV-induced TiO_2_ photocatalysis has been shown to inactivate a wide variety of airborne mammalian viruses, including poliovirus 1, avian and human influenza viruses, and SARS coronavirus, as reported in Table 3. It is worthy of note that any study that did not faithfully show each and every irradiation parameter (i.e., UV dose, irradiation time, or intensity) was deliberately excluded from the survey.

Experimental findings demonstrated that it is possible to inactivate the influenza virus by degrading viral proteins, depending on the UV intensity (I) and the irradiation time (T_irr_) applied during the photocatalysis process. Moreover, the use of photocatalysts has also been exploited with some success for the inactivation of bacteriophages, which were used as model airborne viruses [106,107].

Besides, very recent findings suggested that the use of TiO_2_ nanoparticles in combination with UV-C light was able to efficiently inactivate the human coronavirus HCoV-NL63. Once again, this holds great promises for the inactivation of SARS-CoV-2 [108].

From this literature survey, it is apparent that when using TiO_2_ photocatalysts, an UV-A radiation dose from tenths to hundreds of J cm^−2^ is required to obtain a complete inactivation of airborne bacteria.

## 4. Conclusions and Outlooks

SC-based photocatalytic inactivation of microorganisms is a synergistic bactericidal and virucidal effect of the electromagnetic radiation at a given wavelength and the oxidative radicals produced by the photocatalyst subjected to UV lighting. In this context, TiO_2_ has been extensively investigated and proficiently used in very popular disinfection technologies. The efficiency of the UV-induced TiO_2_ photocatalysis varies as a function of different parameters, namely the crystallinity and the concentration of the photocatalyst, and the proper combination of the intensity of the light applied to the SC and the irradiation time. According to the tables hereinabove, an optimal antimicrobial activity can be reached by exposing a given TiO_2_ surface to UV-A lighting with a radiation intensity in the order of tenths to ten mW cm^−2^ for a time period from 30 min to a few hours, depending on the microorganism. Therefore, UV-A-induced photocatalysis of TiO_2_ holds great promises for the design of atoxic disinfection systems effective against airborne pathogens. On the other hand, far UV-C light *per se*, i.e., without any photocatalyst, has been proposed as a safer option to the use of broad-spectrum UV-C germicidal light for the effective inactivation of both bacteria and viruses. 

In this literature survey, we evaluated and compared the efficacy of UV-based photocatalysis (i.e., TiO_2_ illuminated with UV-A and UV-C) and UV-C alone. Experimental evidence disclosed UV-C as the most effective antimicrobial treatment, while UV-A-based PCO were the least effective technology (i.e., UV-C > UV-C photocatalysis > UV-A photocatalysis) [31,44]. Nonetheless, it has been reported that the exposure of microorganisms to UV-C radiation seldom leads to complete inactivation. Rather, if the dose of UV-C radiation is not sufficient (for instance, <2 J cm^−2^), bacteria can repair some of the DNA damage through light-dependent (photoreactivation) or light-independent (dark repair) mechanisms and become even more resistant to radiation [44,121]. Viewed in this light, other disinfection options are needed.

Because we now spend approximately 90% of our time indoors, confined environments such as offices, classrooms, public transports, hospitals, shops, and restaurants are important contributors to the transmission of airborne diseases. In the midst of the current COVID-19 pandemic, it is more obvious than ever that additional efforts to refine disinfection technologies to mitigate the spread of airborne pathogens are urged. TiO_2_-based photocatalytic technologies and UV lighting systems, together with mitigation strategies, may represent valuable tools in this regard. In the last decades, the research community has put emphasis on the development of more and more efficient TiO_2_ NPs doped with metals, such as silver (Ag) and platinum (Pt) [122], or even non-TiO_2_-based photocatalysts for disinfection, including metal oxides, sulfides, bismuth metallates, graphene-based photocatalysts, carbon nitride-based photocatalysts and natural photocatalysts [123]. Such materials are very promising because they can give rise to visible-light driven photocatalysis, i.e., they broaden the spectrum of radiations that can be used to induce PCO. Unfortunately, the UV irradiation wavelengths used to activate the well-known and widely used TiO_2_ photocatalyst cover only 4% of the sunlight spectrum. Accordingly, the development of visible-light-driven photocatalysts is currently under the spotlight.

## Figures and Tables

**Table 1:** Relevant studies on the photocatalytic activity of TiO_2_ against bacteria and used in the form of nanoparticles and microparticles in suspension.

Target	Photocatalyst	Light Parameters	Irradiation Time	Antibacterial Efficiency	Estimated Minimum UV Dose (According to Equation (1))	Reference
*S. choleraesuis*, *V. parahaemolyticus*, *L. monocytogenes*	10 mg mL^−1^ (Petri dish)	λ = 360 nm (UV-A); I = 0.4 mW cm^−2^	30 min 1 h 1.5 h 2 h	100% at T_irr_ ≥ 2 h	0.3 J cm^−2^ (or W s cm^−2^)	[109]
	0.25–1.25 mg mL^−1^ (batch reactor)	λ = 360 nm (UV-A); I = 0.1 mW cm^−2^	3 h 4 h	100% at T_irr_ ≥ 3 h	0.1 J cm^−2^	
*E. coli*	0.025–1 mg mL^−1^	λ = 400–800 nm (Vis–IR); I = 0.04 mW cm^−2^ I = 0.1 mW cm^−2^	2 h	100% at T_irr_ ≥ 40 min 100% at T_irr_ ≥ 25 min	96 J cm^−2^ 150 J cm^−2^	[110]
*S. aureus,* *S. typhimurium*, *P. aeruginosa*, *E. coli*	1 mg mL^−1^	λ = 368 nm (UV-A); I = n.d.	30 min 1 h 1.5 h 2 h 2.5 h	100% at T_irr_ ≥ 1 h	/	[111]
*E. coli*	1 mg mL^−1^	λ = 310–400 nm (UV-A); I = 0.5 mW cm^−2^	30 min 1 h 1.5 h 2 h 2.5 h 3 h 3.5 h 4 h	100% at T_irr_ ≥ 4 h	1.8 J cm^−2^	[112]
*E. coli*	0.25 mg mL^−1^	λ = 355–375 nm (UV-A); I = 3.6 mW cm^−2^ λ = 254 nm (UV-C); I = 3.6 mW cm^−2^	30 min 1 h 1.5 h 2 h	100% at T_irr_ ≥ 90 min (UV-A) 100% at T_irr_ ≥ 30 min (UV-C)	19 J cm^−2^ (UV-A) 6.5 J cm^−2^ (UV-C)	[44]

**Table 2:** Relevant studies on the photocatalytic activity of TiO_2_-based photocatalyst against bacteria and immobilized onto surfaces.

Target	Photoreactor	Light Parameters	Irradiation Time	Antibacterial Efficiency	Estimated Minimum UV Dose (According to Equation (1))	Reference
*E. coli*	TiO_2_-coated Petri dish	λ = 310–400 nm (UV-A); I = 0.25 mW cm^−2^	2 h 4 h 6 h	100% at T_irr_ ≥ 2 h	7.2 J cm^−2^	[112]
*M. smegmatis*, *B. thuringiensis*	TiO_2_ and Pt/TiO_2_-coated glass	λ = 350–400 nm (UV-A); I = 0.65 mW cm^−2^	10 min 20 min 30 min	99.8% at T_irr_ ≥ 30 min	1.1 J cm^−2^	[113]
*E. coli*	TiO_2_-coated glass	λ = 315–400 nm (UV-A); I = 1 mW cm^−2^	15 min 30 min 1 h 1.5 h	100% at T_irr_ ≥ 30 min	1.8 J cm^−2^	[114]
*E. coli*	TiO_2_-coated filter	λ = 355–375 nm (UV-A) λ = 280–320 nm (UV-B) λ = 254 nm (UV-C); I = 3.6 mW cm^−2^	2 h 4 h 6 h	100% at T_irr_ ≥ 4 h	518 J cm^−2^	[76]
*E. coli*, *P. aeruginosa*, *C. freundii*, *S. aureus*, *S. saprophyticus MRSA*	TiO_2_-coated cellulose acetate monoliths	λ = 365 nm (UV-A); I = n.d.	5 min 10 min 15 min 20 min	100% at T_irr_ = 20 min	/	[77]
*E. coli*	TiO_2_ film	λ = 365 nm (UV-A); I = n.d.	1 h 3 h 6 h 8 h	100% at T_irr_ ≥ 6 h	/	[115]
*E. coli*	Continuous annular reactor with TiO_2_-coated filter	λ = 365 nm (UV-A); I = 0.5 mW cm^−2^ I = 3.4 mW cm^−2^	1.1 min	100%	0.03 J cm^−2^ 0.204 J cm^−2^	[79]

**Table 3:** Relevant studies on the photocatalytic activity of TiO_2_-based photocatalyst against viruses.

Target	Photoreactor	Light Parameters	Irradiation Time	Antiviral Efficacy	Estimated Minimum UV Dose (According to Equation (1))	Reference
Influenza virus H1N1	TiO_2_-coated porous ceramic substrate	λ = 365 nm (UV-A); I = 1 mW cm^−2^	4 min 10 min 15 min 30 min	100% at T_irr_ ≥ 5 min	0.3 J cm^−2^	[74]
Vaccinia virus, influenza virus H3N2	TiO_2_ and Pt/TiO_2_-coated glass	λ = 350–400 nm (UV-A); I = 0.65 mW cm^−2^	10 min 20 min 30 min	99.8% at T_irr_ ≥ 30 min	1.1 J cm^−2^	[113]
Influenza virus H1N1	TiO_2_-coated glass	λ = 352 nm (UV-A); I = 0.001 mW cm^−2^ I = 0.01 mW cm^−2^ I = 0.1 mW cm^−2^ I = 1 mW cm^−2^	2 h 4 h 6 h 8 h	100%	0.8–14.4 J cm^−2^	[116]
HSV-1 virus	TiO_2_ film	λ = 365 nm (UV-A); I = n.d.	6 h	100%	/	[115]
Noravirus	TiO_2_ photocatalytic reactor	λ = 254 nm (UV-C)	4 min 10 min 15 min 20 min	100% at T_irr_ ≥ 10 min	2.7 J cm^−2^	[117]
Qβ and T4 bacteriophages	TiO_2_-coated glass	λ = 351 nm (UV-A) I = 0.001 mW cm^−2^ I = 0.01 mW cm^−2^ I = 0.1 mW cm^−2^	4 h 8 h 24 h	99.99% at T_irr_ ≥ 8 h (with I = 0.1 mW cm^−2^)	28.8 J cm^−2^	[106]
Influenza virus H1N1, Enterovirus type 71	1% wt Ag/TiO_2_-coated glass	λ = 365 nm (UV-A)	20 min	99.99% at T_irr_ = 20 min	/	[118]
Human norovirus	Cu/TiO_2_ nonwoven fabric	λ = 365–405 nm (UV-A); I = 5000 mW cm^−2^	1–60 min	99% at T_irr_ ≥ 48 min	/	[119]
T4 bacteriophage	TiO_2_-coated βSiC foam	λ = 392 nm (UV-A); I = 11.7 mW cm^−2^	15 min 30 min 45 min 60 min	99.9% at T_irr_ ≥ 60 min	42.12 J cm^−2^	[107]
Avian influenza virus H9N2	TiO_2_-coated Petri dish	λ = 365 nm (UV-A); I = 0.5 mW cm^−2^; I = 1 mW cm^−2^; I = 1.5 mW cm^−2^	30 min 1.5 h 2.5 h	100% at T_irr_ = 2.5 h	4.5 J cm^−2^	[120]
Human coronavirus HCoV-NL63	TiO_2_-coated glass	λ = 254 nm (UV-C); I = 2.9 mW cm^−2^; I = 4.3 mW cm^−2^; I = 13 mW cm^−2^	1 min 5 min 10 min	100% at T_irr_ = 1 min (with I = 2.9 mW cm^−2^)	0.17 J cm^−2^	[108]

## Data Availability

The data presented in this study are openly available in Scopus.
